# Biodiversity of Indonesian indigenous buffalo: First review of the status, challenges, and development opportunities

**DOI:** 10.14202/vetworld.2023.2217-2229

**Published:** 2023-11-02

**Authors:** Peni Wahyu Prihandini, Yuli Arif Tribudi, Dwi Nur Happy Hariyono, Aprilianna Putri Zahara Nafsina Luvita Sari, Lisa Praharani, Eko Handiwirawan, Bess Tiesnamurti, Endang Romjali, Procula Rudlof Matitaputty, Agus Wiyono

**Affiliations:** 1 Research Center for Animal Husbandry, Research Organization for Agriculture and Food, National Research and Innovation Agency, Cibinong, Bogor, 16915, Indonesia; 2 Department of Animal Science, Faculty of Agriculture, Universitas Tanjungpura, Pontianak, 78121, Indonesia; 3 Department of Animal Science, Faculty of Agriculture, Universitas Khairun, Ternate, 97719, Indonesia; 4 Department of Animal Breeding and Reproduction, Faculty of Animal Science, Universitas Gadjah Mada, Yogyakarta, 55281, Indonesia; 5 Research Center for Veterinary Science, Research Organization for Health, National Research and Innovation Agency, Cibinong, Bogor, 16915, Indonesia

**Keywords:** *Bubalus bubalis*, diversity, production system, zoogenetic resources

## Abstract

In Indonesia, the buffalo is important for small and marginal farmers’ livelihood and economic development as a source of food, working animal, and tourist attraction. Therefore, an in-depth study is needed to examine challenges and opportunities for buffalo development in Indonesia. In Indonesia, the buffalo is divided into two types: swamp buffalo and river buffalo. The buffalo population in Indonesia has declined significantly. A decrease of approximately 39.35% was recorded from 2022 to 2017. The decline occurred due to low reproduction rate and suboptimal rearing management systems. There are three buffalo-rearing systems: Intensive, semi-intensive, and extensive. The productivity of buffalo is diverse and closely related to the characteristics of the regional agroecosystem, consistent with existing natural resources and rearing management systems. The diversity of buffalo productivity provides a good opportunity to improve productivity. Improvement of buffalo genetics is urgently needed, by improving mating management, etc., especially to reduce potential inbreeding. In recent years, genetic and molecular research on Indonesian buffalo has made progress, including use of molecular markers, such as microsatellites and single-nucleotide polymorphisms, to evaluate genetic diversity within and among buffalo populations across Indonesia. In addition, studies are being conducted on the relationship of genotype mutations that contribute to appearance and phenotypic performance (heat stress, reproduction, behavior, coat color, and production attributes) in buffaloes. Identification of genetic diversity in local buffaloes can be improved using various genetic and genomic techniques. These findings will form a basis for the targeted conservation of local buffaloes in Indonesia. This study aimed to collect information on the genetic resources of the local buffalo, particularly its status and production system and provide recommendations for developing buffalo production in Indonesia.

## Introduction

Indonesia has one of the largest livestock populations in Asia, contributing greatly to international livestock biodiversity. Estimates from the Central Statistical Agency of Indonesia indicate that the country has approximately 18,053,710 beef cattle, 578,579 dairy cattle, 1,189,260 buffalo, 394,341 horses, 19,397,960 goats, 15,615,300 sheep, 314,101,311 birds (indigenous chicken), and 49,876,959 ducks [1]. Among many livestock species, the buffalo makes an important contribution to the livelihood of a large proportion of small and marginal farmers by providing meat and milk as a source of high-quality protein, dung as fuel and organic fertilizer, mechanical or draught power, and hides and skins as raw materials for industry. They are also kept by farmers for social, religious, and cultural purposes. In Indonesia, buffaloes are generally classified into Murrah (river buffalo) and swamp buffalo (*Bubalus bubalis*). They spread in various agroclimatic conditions, which has led to the development of various breeds or strains adapted to specific environmental conditions. In Asia, the river buffalo accounts for approximately 75% of the total buffalo population, mainly in South and West Asia, with the remaining 25% being the swamp type found in Southeast Asia and Southern China [2, 3]. Approximately 90% of the buffaloes in Indonesia are swamp buffaloes raised for draught power and meat production. Nonetheless, according to the use made of these two subspecies across Asia, a different growth trend has been reported: (i) A positive trend in the buffalo population in milk-producing countries of South Asia and (ii) a dramatic decline in many Southeast Asian countries where buffaloes are used mainly as draft animals [4].

Studies on the scope and prospects of buffalo production in Indonesia are lacking. Their contribution to the agricultural sector and potential for economic development have not been adequately examined or comprehensively addressed in academic research. This has resulted in a limited understanding of the scope and opportunity associated with buffalo production, preventing their full potential from being realized in Indonesia’s agricultural landscape. Therefore, extensive studies must aim to close this knowledge gap, emphasizing the importance of buffaloes, and developing strategies that effectively utilize their advantages.

This study aims to fill the gap on the genetic resources of indigenous buffalo in Indonesia, especially their status, production system, and opportunities, as well as provide recommendations for buffalo production in Indonesia.

## Domestication of Buffalo and its Dispersal in Indonesia

Macgregor [5] first recognized two types of water buffaloes native to Asia, based on morphological and behavioral features – river and swamp types. The river breeds include Mullah, Nirirabi, and Surti in India and Nirirabi and Kundi in Pakistan [6]. The river buffalo is native to the Indian subcontinent and has spread westward to the Balkans, Greece, Egypt, and Italy, whereas the swamp buffalo is found throughout Southeast Asia – from Assam and Bangladesh in the west to the Yangtze River Valley in China [7, 8]. River buffalo makes up almost 75% of the Asian buffalo population. Molecular and morphological evidence indicates that swamp buffalo populations have strong geographic genetic differentiation and a lack of gene flow, but strong phenotypic uniformity [9]. In contrast, river buffalo populations show a weaker phylogeographic structure, but higher phenotypic diversity [8, 10]. Molecular evidence, as well as archeological evidence by Nagarajan *et al*. [11], have indicated that the river buffalo was domesticated in an atypical manner, involving continuous introgression of wild animals into domestic stocks in the Indian subcontinent before the mature phase of the Indus Valley civilization (2600–1900 BC), whereas the swamp buffalo was domesticated in a region far south of China, northern Thailand, and Indochina. Following domestication, it spreads south through peninsular Malaysia to Sumatra, Java, and Sulawesi, and north through China to Taiwan, the Philippines, and Borneo [12]. Cockrill [13] suggested that the swamp buffalo was domesticated in the Yangtze Valley approximately 4000–5000 years ago. Recently, molecular analysis using complete mitochondrial D-loop sequences suggested the Lower Yangtze as the first domestication center of the swamp buffalo, followed by migration into two different routes: one along the Upper Yangtze to Southwest China and the other to Southeast China and then to Southeast Asia through Guangxi province [14].

The buffalo is associated with religious sacrifice in Indonesia, especially in rural areas – evidenced by their discovery at ancient ruins and persisting culture and beliefs in Baan *et al*. [15] Toraja-South Sulawesi, Sumba-Nusa Tenggara East, Sumbawa-West Nusa Tenggara, Minangkabau, West Sumatra, Pampangan-South Sumatra, etc., where they have become a tourist attraction. Buffalo breeding is ingrained in society since the reign of Indonesian kings [16].

## Buffalo Breeds in Indonesia and their Importance

**Figure-1:**
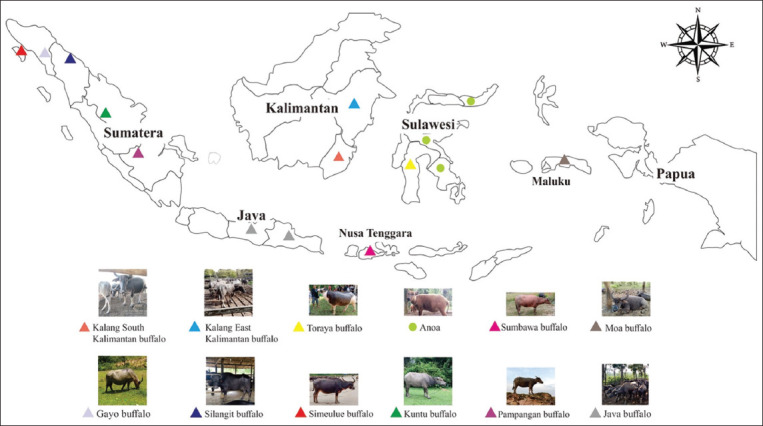
A map showing the distribution of buffaloes in Indonesia [Source: https://www.cleanpng.com/png-flag-of-indonesia-vector-map-indonesia-752719/].

The buffalo population in Indonesia almost entirely (90%) comprises swamp buffaloes, which have thrived due to remarkable adaptability in acclimatizing to various environments, resulting in the development of a breed that is well-suited to the agroecological characteristics of its habitat. Their adaptability to a wide range of ecological conditions, including wetlands, marshes, and other similar settings [15], has allowed them to thrive in practically every region of the country. According to the Domestic Animal Diversity Information System [17], buffalo breeds in Indonesia consist of Anoa, Gayo, Jawa, Kalang South Kalimantan, Kalang East Kalimantan, Kuntu, Moa, Murrah, Pampangan, Simeulue, West Sumatra, North Sumatra, Sumbawa, and Toraya (Table-1 [17] and Figure-1). Some live on small islands with a closed population of other breeds, such as Moa buffaloes. Their adaptation to their habitats has enabled the evolution of distinct traits and characteristics that correspond to the agroecological conditions of their environments [8, 18]. For example, the buffaloes of Pampangan, South Kalimantan, and East Kalimantan have adapted to the environment of swamps, lakes, and rivers, respectively. Sumbawa buffaloes have adapted to savanna areas with droughts that last for more than 8 months a year. Simeulue buffaloes have adapted to coastal and hilly areas and Toraya to agricultural areas [19, 20].

**Table-1:** List of buffalo breeds present in Indonesia and their estimated risk level from the FAO-DAD-IS database [17].

Breed	Alternative breed name	Use of the breed	Risk level	Localization and distribution
Anoa	None	Culture	Unknown	Sulawesi
Gayo	Koro	Meat producer, social, culture, labor, and economic uses	Not at risk	Gayo Lues districk, Aceh Province
Java	Kebo	Draught power	Unknown	Java
Kalang South Kalimantan	None	Draught power, fertilizer, meat, and race	Not at risk	In almost all districts in South Kalimantan, Central Kalimantan, and also spreaded in East Kalimantan
Kalang East Kalimantan	None	Meat, fertilizer, and tourist attraction	At risk	Only in Kalimantan, especially in South and Central of Kalimantan where the area is swampy
Kuntu		Meat, hobby, and religious ceremonies	Not at risk	Kampar Kiri District
Moa	Oriory	Meat and milk	Not at risk	Moa Island and a number of districts in Maluku; and spread to NTT, NTB, East Java, South Sulawesi, Papua and East Timor
Murrah	None	Milk	Unknown	North Sumatra, Central Java, East Java
Pampangan	Kerbau Lumpur	Draught power, meat, milk, fertilizer, and religious ceremonies	At risk	Especially Pampangan Subdistrict, then spread to Subdistricts of Jejawi, Lampam Pangkalan, Air Sugihan, Tulung Selapan and Pedamaran as well as to Districts of Banyuasin and Ogan Ilir
Simeulue	Kerbau Gunung	Meat, milk, saving security, fertilizer, hobby, and religious ceremonies	Not at risk	Salang region, West Simeulue, Central Simeulue district, and Alafan district
West Sumatra	Kerbau Lumpur	Meat, milk, and fertilizer	Unknown	West Sumatra
North Sumatra	Kerbau Lumpur	Meat, milk, fertilizer, and draught power	Unknown	North Sumatra
Sumbawa	None	Meat, milk, saving security, hobby, and religious ceremonies	Not at risk	Mostly in NTB, and spread throughout the islands of Nusa Tenggara and various provinces in Indonesia; even to Timor-Leste
Toraya	Tedong Bonga, Kerbau Belang	Meat, transport, religious ceremonies, and tourist attraction	Not at risk	Tana Toraja and North Toraja

Buffaloes play a strategic role in the lives of the Indonesian people. Buffalo is not only a source of food but also functions as a working animal, producing organic fertilizer and biogas, which is specially useful for agricultural areas. In addition, buffaloes play an important role in sociocultural (tradition and rituals), religious, and recreational or sporting activities [20, 21]. Buffaloes play important symbolic roles in Indonesia, such as in Toraja-South Sulawesi, Sumba-East Nusa Tenggara, Sumbawa-West Nusa Tenggara, Minangkabau-West Sumatra, and Pampangan-South Sumatra. They have developed into tourist attractions globally [19]. The variations in regional demand of this species have influenced the use of different buffalo genetic resources.

## Buffalo Population in Indonesia

The Indonesian government has made several efforts to protect its local livestock species and breeds. The Indonesian government has established site-specific buffalo breeds based on the Decree of the Indonesian Minister of Agriculture. The determination of local livestock breeds refers to the Regulation of the Minister of Agriculture Number: 19/Permentan/OT.140/2/2008 concerning the Registration and Release of Livestock Breeds or Lines, which has been revised to the Regulation of the Minister of Agriculture Number: 117/Permentan/SR.120/10/2014.

**Figure-2:**
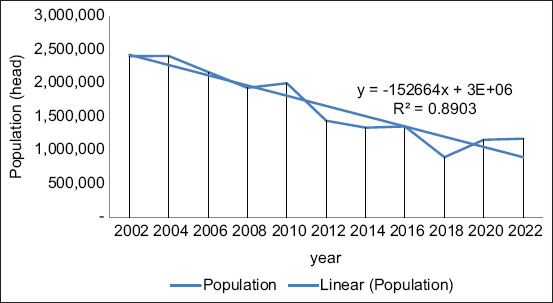
Trend of buffalo population in Indonesia from 2002 to 2022 [22].

Buffaloes are frequently utilized as draft animals to plow paddy fields and for other farming-related activities and are an essential part of Indonesia’s agricultural sector. Despite their substantial importance to the national economy, there have been growing concerns about the decrease in buffalo population. According to data from the Directorate General of Livestock and Animal Health, Indonesian Ministry of Agriculture [22], the buffalo population in 2002 was 2.4 million, which declined to 1.17 million in 2022. The trend for buffalo population in Indonesia was negative (*y* = −152664*x* + 3E + 06, *R*^2^ = 0.8903) (Figure-2) [22]. A sharp decline in the buffalo population occurred from 2010 to 2012 (−28%) and from 2016 to 2018 (−34%). Hegde [23] reported a −39.35% reduction in the Indonesian buffalo population from 2002 to 2017, the third largest population decline after Sri Lanka (−57.04%) and Thailand (−44.67%). Factors such as limited feed during dry season, conversion of grazing lands, and long periods of rearing [24] have contributed to this decline and the increasing popularity of motorized farming equipment [8]. Ernest *et al*. [25] discovered that habitat fragmentation, loss, and limited genetic diversity are key drivers of the decrease in buffalo population. Metzger *et al*. [26] and Mureithi *et al*. [27] emphasized the importance of community-based conservation activities in reducing the decline in buffalo populations.

**Figure-3:**
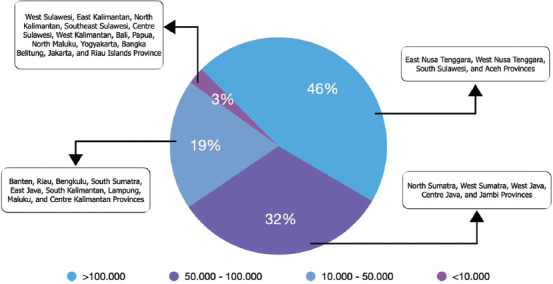
Population distribution of buffalo based on the data of 2022 [22].

Figure-3 [22] shows the distribution of buffaloes in 37 provinces in Indonesia with different population densities. The distribution of buffalo populations is considerably affected by geographical and climatic conditions and cultural and economic factors. Based on the population data of 2022 [22], most buffaloes (46%) are found in the provinces of East Nusa Tenggara (172,850), West Nusa Tenggara (129,037), South Sulawesi (118,074), and Aceh (117,317), with a total population of >500,000. Other provinces with significant buffalo populations include North Sumatra, West Sumatra, West Java, Central Java, and Jambi, contributing 32% to the total buffalo population (population of each province in the range of 50,705–98,246), whereas the other provinces account for 21%. However, no information is available on buffalo distribution based on the type or breed among Indonesia’s reported 1.17 million buffaloes. This lack of information poses challenges for effective breeding and management practices, because different breeds may have distinct requirements and characteristics.

## Buffalo Breeding System in Indonesia

Specific buffalo breeds are raised in specific agroecosystems (lowland, midland, and highland). For instance, the Simeulue, Kuntu, Sumbawa, Moa, and Silangit buffaloes are raised in lowlands; Silangit in oil palm and rubber plantations; Toraya in midlands; Gayo in highlands; and Pampangan in lowland, swampy areas.

Kalang buffalo, a local buffalo that is geographically distributed along the river from East to South Kalimantan, is a regional biodiversity resource. Kalang buffalo can thrive in swamplands [28].

In Indonesia, the number of buffalo owned per farmer is relatively low (Table-2): 1–7 in Aceh [29] and 4–10 in West Sumatra [30]. However, some farmers own relatively large numbers (>50 heads per farmer) of buffalo, for example, in Gayo, Silangit, Pampangan, Kalang South Kalimantan, and Sumbawa. The ownership of Simeulue, Kuntu, Kalang East Kalimantan, and Moa buffaloes ranges from 10 to 50 per farmer. Fewer than ten of Toraya buffaloes are owned per farmer. This small number has a significant influence on the development of management systems for buffaloes. A survey conducted by Kartika *et al*. [31] in South Sulawesi showed that the five main factors that contribute to the low numbers include limited land for feed, low capital, limited labor, lack of bulls, and low fertility rate.

**Table-2:** Buffalo management practiced by the farmers.

Breed	Management types	Mating system	Agroecosystem	Ownership (head)
Simeulue	Extensive	Natural	Lowland	1–23
Gayo	Extensive	Natural	Upland	1–59
Silangit	Semi-intensive	Natural	Lowland (integrated with oilpalm)	14–110
Kuntu	Extensive	Natural	Lowland	1–30
Pampangan	Semi-intensive	Natural	Lowland (swamp)	3–84
Kalang South Kalimantan	Semi-intensive	Natural	Lowland (river)	5–100
Kalang East Kalimantan	Semi-intensive	Natural	Lowland (river)	20–30
Sumbawa	Semi-intensive	Natural	Lowland	2–119
Toraya	Intensive	Natural	Middleland	1–5
Moa	Extensive	Natural	Lowland	10–40

The 10 buffalo breeds observed had three types of management systems: Intensive, semi-intensive, and extensive (Table-2). Toraya buffaloes are kept in intensive systems; Silangit, Kalang South Kalimantan, Kalang East Kalimatan, Pampangan, and Sumbawa in semi-intensive systems; and Simeulue, Gayo, Kuntu, and Moa in extensive systems. The Kalang East and South Kalimantan buffaloes are raised on the Kalang system (a wooden structure on the river) [32]. In the intensive system, buffaloes are kept in barns. In the semi-intensive system, buffaloes are kept in the barn at night and grazed on the pasture during the day. The grazing activities start in the morning and last the afternoon. Every morning, the keeper or farmer opens the barn door and lets the buffaloes out into the pasture to graze and wallow. During the night, forages are provided in the barn. During the dry season, buffaloes are grazed all day in the paddy field after harvest and then sleighed to the river or swamp area to wallow. In the rainy season, buffaloes are kept in the Kalang at night.

The feeding system can be grouped into three types: fully grazing (extensive), cut and carry (intensive), and grazing during the day and feeding at night (semi-intensive). Simeulue, Gayo, Kuntu, and Moa graze on the pasture all day. Sumbawa and Pampangan graze on the pasture, whereas Silangit graze in oil palm and rubber plantations. Silangit buffaloes are given concentrate feed and additional minerals every day to improve milk production. Farmers provide additional feed at night, such as tofu byproduct, palm oil cake, and sweet potato byproduct. Kalang South and East Kalimantan buffaloes were grazed on swamp land according to their habitat. The types of forages available are local and natural grasses. Forage availability is influenced by the season in South Kalimantan and is limited [33]. The feeding system of Toraya buffaloes is intensive.

According to Rusli *et al*. [34], Gayo buffaloes can develop because of abundant land and natural resources, such as agricultural waste, are available. Pampangan buffaloes are grazed on swamp land. Kalang East and South Kalimantan buffaloes are grazed on swampland during the day and provided forages, such as Kumpai grass (*Hymenachne amplexicaulis* [Rudge] Nees) at night in the Kalang barn, with no additional concentrate or supplement [35].

Toraya buffalo is highly priced because it is related to local culture. Toraya buffaloes are fed cultivated grasses, such as elephant grass, Taiwan elephant, and Setaria spp., and agricultural byproducts (corn, rice, and soybeans) from their own fields. Moa buffaloes graze on natural grasses, and it seems that no improvement in pasture management has occurred, which still relies on natural vegetation.

## Challenges in Buffalo Farming

Buffaloes play strategic roles in the Indonesian community. Buffalo meat is considered a complement or substitute to beef, thus emphasizing its essential role in supplementing national protein needs [36]. Buffalo milk significantly impacts human nutrition because it contains more essential nutrients than cow milk. Individuals with bovine milk allergies can consume buffalo milk without experiencing adverse reactions. Buffalo milk has elevated levels of lipids, lactose, protein, casein, and arsenic [37, 38]. Although the cholesterol content of buffalo milk is lower than that of cow milk, it delivers more energy per unit volume of milk and is a rich source of iron, phosphorus, vitamin A, and tocopherol (a natural antioxidant). However, unlike raw cow milk, raw buffalo milk is not widely consumed [39, 40].

Buffaloes are mainly used in farming activities due to their strong ability to plow rice fields [41]. However, due to increased farming mechanization, buffaloes have experienced a decline in value and production (−4.92%) over the past two decades [42], and their use has shifted to meat and milk production. The demand for buffalo meat has increased in Indonesia, leading to the development of buffalo production, although challenges exist, such as decreased grazing land, low productivity, poor access to investors, and low adoption of technology.

Traditional agricultural practices contribute to low productivity because buffaloes are released on land that has been used since the livestock existed. Natural feed is still relied upon, and it is highly uncommon for breeders to use plant forage reserves for animal feed. The increase in the buffalo population is insignificant because breeders that practice such maintenance systems pay little attention to animal health, rely on natural mating, and use inefficient recording. The inbreeding rate in the population is very high because breeding programs are lacking [29, 43]. According to Rusdiana *et al*. [44], the mortality rate of buffaloes is high. Buffaloes spend most of the day grazing and return to the stables at night, limiting the farmers’ ability to monitor their health.

Buffaloes are slow-breeding animals. According to Budiarto and Ciptadi [45], in Malang regency, male and female swamp buffaloes attained sexual maturity at 2 and 2–2.5 years, respectively. With a long gestation (10–11 months), the first birth is at 40 months. The leading cause of low buffalo productivity is the adult female buffalo’s lack of intense maintenance, lack of recording, and occurrence of silent heat. As a result, reproduction management becomes inefficient. Inefficient feeding nutrition and lack of feed availability also impact reproductive and productive performance by delaying the resumption of the estrous cycle, increasing calving interval, and reducing growth rate [4]. Despite breeding inefficiencies, keeping even small populations of buffaloes (2–10 animals) is expected to contribute to the development of buffalo farming in Indonesia.

A widespread problem in the field of livestock in Indonesia is a lack of education for breeders, which restricts technological adaptation and knowledge development, notably in buffalo farming. According to Nafianda *et al*. [46], the level of education of the breeders influences their intake of information and outlook. Farmers who are uneducated find it difficult to adopt new technologies. However, these challenges will be overcome gradually due to the breeders’ experience and the extension operations that have been implemented. However, when raising livestock is a secondary occupation, the condition becomes worse. Furthermore, Indonesia does not have a designated institution for buffalo development that aims to improve production and reproduction performance, by providing a centralized platform for research, knowledge dissemination, and technical support [47].

In Indonesia, buffalo milk is processed into products similar to yogurt, cheese or processed buffalo milk using bacteria and extracted vegetables (wild eggplant), fruit (orange, pineapple), or leaves (papaya leaves) (Table-3) [48–52]. The products were found in several provinces with different local names. These products have high nutritional value, such as high protein and low fat. Wirawati *et al*. [48] reported that the nutritive value of dadih from West Sumatra had 5.01%–7.06% protein; 6.5%–8.17% fat, 0.68%–0.92% ash, pH 4.47–4.8, and 1.17%–2.28% total titratable acidity. Sulmiyati and Said [49] reported that dangke has a nutritive value within the following ranges: curd production 41.38%–52.20%, pH 6.92–6.96, and lactic acid 0.15%–0.70%. This study revealed that the best quality of dangke from buffalo milk was derived with the addition of 1% crude papain. The physicochemical characteristics of our dangke were as follows; 43.94% curd, 51.14% whey, pH 6.96, and 0.15% lactic acid. Thus, farmers can benefit from processing buffalo milk into traditional cheese using locally available ingredients, such as crude papain.

**Table-3:** The products of buffalo milk in Indonesia.

Product name	Product type	Added ingredient	Province	References
Dadih	Cheese	*Lactobacillus* spp.	West Sumatra	[48]
Dangke	Cheese	Crude papain	South Sulawesi	[49]
Dali ni horbo	Cheese	Extract papaya leave, pineapple	North Sumatra	[50]
Palopo	Processed buffalo milk	Soy milk, extract of wild eggplant	West Nusa Tenggara	[51]
Keju Moa	Cheese	Extract papaya leave	Maluku	[52]

Several studies have been conducted to investigate the probiotic activity of lactic acid bacteria in buffalo milk in West Sumatra province [53] and North Sumatra [54] using buffalo milk of dairy buffalo breeds, as well as the milk of the Tedong Bonga breed in South Sulawesi [55]. These investigations are of interest because researchers are trying to determine the potential of buffalo milk of different breeds in various provinces.

Susmiati *et al*. [56] reported that buffalo milk in West Sumatra was also processed into a probiotic beverage using *Lactiplantibacillus pentosus* HBUAS53657 and 20% sweet orange juice. Similarly, farmers on Moa Island in Maluku province processed milk into a beverage using papaya leaves. At least two studies [57, 58] have reported the use of dadih and dali ni horbo as food additives for children to prevent from stunting. The result showed a correlation between body weight and dadih or dali ni horbo consumption in children. In addition, children with lactose intolerance could consume local buffalo milk. This finding is of interest because local government can provide food substitutes for children by utilizing local products. However, more studies and longer periods of observation are required to confirm the correlation between dadih consumption and growth in children.

## Development of Genetic Studies in Indonesian Indigenous Buffalo

Compared to other livestock species, research interest in buffalo, especially buffalo genetics, is limited. The potential for developing indigenous buffalo is not widely recognized or appreciated, leading to a lack of interest in this animal. Many buffalo studies have been limited to the characterization of breeders in a specific location [59, 60] or the performance characterization of a specific buffalo breed [35, 43, 61].

In recent years, genetic and molecular research on Indonesian buffaloes has advanced, offering valuable insights into their genetic diversity and potential for selective breeding [62]. Genetic diversity is crucial for the survival and adaptability of a species [63]. Recent studies have focused on assessing the genetic diversity of Indonesian buffalo populations to ensure their conservation and sustainable utilization. Researchers have utilized molecular markers, such as microsatellites and single-nucleotide polymorphisms, to evaluate the genetic diversity within and among buffalo populations across Indonesia [62, 64].

**Figure-4:**
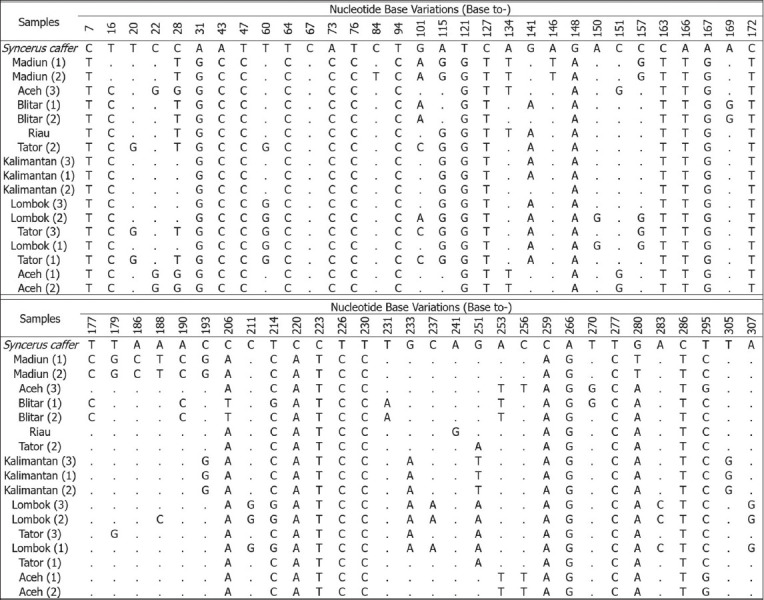
The variations of nucleotide base of cyt *b* gene sequences of the Indonesian *Bubalus bubalis* and the outgroup (*Syncerus caffer*) [67].

**Figure-5:**
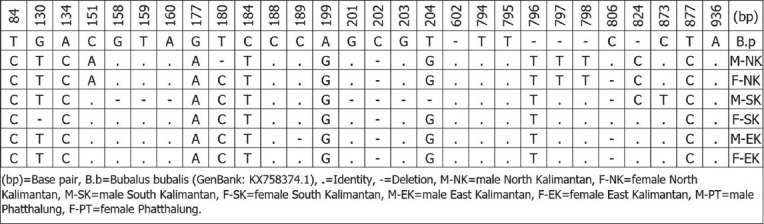
The variations of nucleotide base of d-loop gene sequences of the Indonesian *Bubalus bubalis* [69].

**Figure-6:**
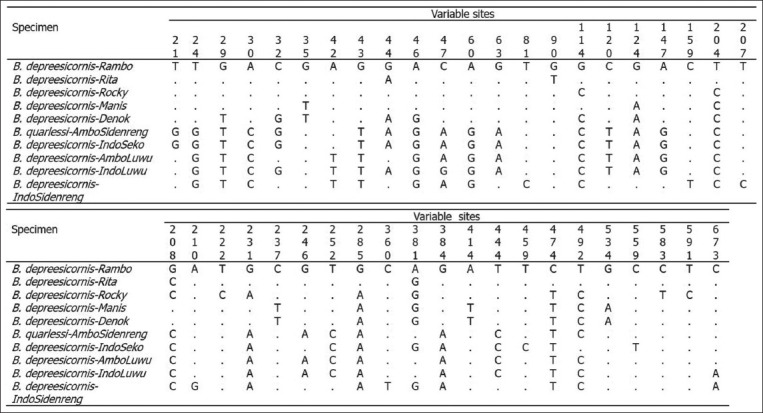
The variations of nucleotide base of COI gene sequences of the Anoa, a dwarf buffalo of Indonesia [70].

**Figure-7:**
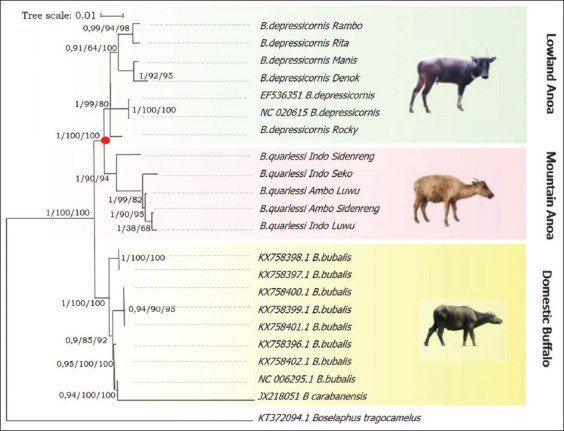
Reconstruction of the Anoa phylogenetic tree [70].

Genetic diversity analysis based on mitochondrial DNA (mtDNA) from Indonesian buffalo populations has been studied; The evolution of mtDNA occurs by the pair substitution of a single base, and the rate of mtDNA evolution is approximately 10 times faster than that of nuclear DNA [65, 66]. According to Sukri *et al*. [67] and Rusdin *et al*. [68], sequence alignment of the cyt *b* gene in Indonesian buffalo populations showed that the T→C transition (silent substitution) was more prevalent than transversions. The mutation sites reported by Sukri *et al*. [67] are shown in Figure-4 [67]. The mtDNA D-loop gene of Indonesian buffalo was reported in a study by Suhardi *et al*. [69]. Alignment of mtDNA D-loop sequences of Kalang and Kayan buffaloes from Kalimantan with those of buffaloes from Thailand revealed 24 haplotypes with several mutations, including transitions (293), transversions (60), deletions (15), and insertions (20) (Figure-5 [69]). Priyono *et al*. [70] showed highly variable sites of COI genes in Anoa, a dwarf buffalo from Sulawesi (Figure-6 [70]). Phylogenetic tree analysis, based on COI gene sequences, showed two types of Anoa in separate clades (Figure-7 [70]).

Neighbor-joining analysis distinguished the samples into two subclades, but all were present in one cluster compared with *Bison bison* [71]. The phylogenetic tree constructed by Winaya *et al*. [72] showed that the most plausible route of buffalo domestication in Indonesia began in mainland China and then split into two branches in the Philippines and Malaysia. The domestic buffalo population in Indonesia is divided into two major clusters: western Indonesian *B. bubalis* (Aceh, Madiun, and Blitar) and central-eastern Indonesian *B. bubalis* (Kalimantan, Sulawesi, and Lombok). Consistently, Rusdin *et al*. [68] reported that cluster one contained Aceh, North Toraja, West Nusa Tenggara, Banten, Kolaka, and Konawe populations and cluster two contained Bombana Island, Bombana mainland, Kolaka, and Konawe populations. Phylogenetic studies based on mtDNA sequences revealed that the buffalo population groupings have a genetic link corresponding to their biogeographic origin. These studies revealed significant genetic variation, highlighting the need appropriate conservation strategies to maintain diversity and prevent the loss of unique genetic resources.

The fundamental problem in genetics is determining the significance of genetic variants in and their physiological effects on phenotypic features [73, 74]. Various studies have been conducted to investigate the relationship between genotypic mutations that contribute to phenotypic appearance and performance (heat stress, reproduction, behavior, coat color, and production attributes) in buffaloes, especially in India [75–78]. However, in Indonesia, reports regarding nucleotide variants of economical candidate genes are lacking.

The growth hormone (GH) gene controls growth performance in livestock. Sumantri *et al*. [79] revealed that the GH/MspI and GHRH/HaeIII loci were polymorphic, whereas the GH|AluI, GHR|AluI, and Pit-1|HinfI loci were monomorphic. In Simeulue buffalo, only GHRH/HaeIII was polymorphic, but did not significantly influence (*P* > 0.05) body size characteristics [80]. Polymorphisms in GH/MspI and GHRH/HaeIII loci were found in swamp buffaloes in Kabaena Island, Bombana District [81]. For a different gene, no polymorphisms were detected in exon 6 Pit-1|Hinf1 in four populations of Indonesian buffaloes (Banten, North Sumatra, Central Java, and West Nusa Tenggara) [82].

For milk production, the amplified fragments were restricted with EcoRV, which cuts the k-casein gene in exon 4 at GAT|ATC, revealing one polymorphism at base position 23, which occurs with a substitution of Ile (ATC) of the T genetic variant into Thr (ACC) of the C genetic variant. In the river buffalo, the k-casein/EcoRV loci produced three genotypes (CC, CT, and TT), whereas in the swamp buffalo, it produced one genotype (CC). This study showed that k-casein|EcoRV were polymorphic in riverine buffalo and monomorphic in swamp buffalo [83]. The study of CSN3 gene reported by Margawati *et al*. [84] showed that all samples possessed BB genotypes, which merged the pattern of the digested fragments with sizes of 255 and 154 bp. This finding suggests that genotyping using HindIII reveals monomorphic BB types associated with milk content of casein.

Regarding morphological characteristics, Indonesian buffaloes possess one of the most unique physical characteristics, for example, characteristics of the white-spotted buffalo are only found in the Carabao breed from the Philippines and Fuling breed from China [85]. The Toraja people of South Sulawesi, Indonesia, place a high cultural value on this breed, which they call Tedong Bonga. Toraja people use Tedong Bonga for customary ceremonies, such as funerals, and as a sign of prosperity and social rank, in contrast to other buffaloes employed to plow paddy fields or produce meat and milk. Therefore, Tedong Bonga is quite valuable economically [86]. Yusnizar *et al*. [87] identified two independent loss-of-function mutations that cause white-spotted coat color in swamp buffaloes: a premature stop codon (c.328C > T, p.Arg110*) and a donor splice-site mutation (c.840 + 2T > A, p.Glu281_Leu282Ins8). The nonsense mutation leads to a premature stop codon in exon 3, and likely removes the mRNA through nonsense-mediated decay pathway, whereas the donor splice-site mutation leads to aberrant splicing of exon 8, which encodes part of a highly conserved region of MITF.

## Opportunities for Genetic Improvement

Almost all buffaloes in Indonesia are used for producing meat and <1% for producing milk (this is a cross between local buffalo and Murrah and Nilli Ravi buffalo). In Indonesia, buffalo farming is predominantly based on smallholder farms and plays a key role in supporting the livelihoods of millions in rural areas. In general, buffaloes are raised in a traditional system. The buffaloes grazed range land during the day, without rotation and adjustment of land holding capacity, and kept in the barn during the night, without additional feed.

The lack of available male buffaloes and undirected mating management may have led to inbreeding. Buffalo mating occurs randomly over several generations, increasing the inbreeding rate by 10%–30% in each generation. Albinoid color, few or many white stripes, horns (one or both) pointing downward, stubby tail, and blue eyes are indicators of inbreeding [19].

Farmers select buffaloes to keep based on their outward appearance. However, some farmers keep buffaloes with high selling price for traditional events, such as in Tana Toraya, South Sulawesi, where farmers select specific colors and patterns. Here, buffalo selection is unrelated to production criteria (body weight or daily gain). In general, farmers do not conduct targeted selection and mating programs for buffalo-rearing; therefore, buffalo productivity in the field is highly varied. These conditions can increase productivity by improving genetic quality through selection and improvement of the mating system.

Genetic quality can be improved through reduced inbreeding, by providing superior bulls at the location and rotating bulls between groups of female buffaloes. For outbreeding programs, the genetic distance between buffalo populations within a province can be estimated by analyzing morphology and DNA markers [68, 71, 88–90]. If applied consistently, this approach can gradually reduce the inbreeding level in Indonesia’s buffalo population. Superior male buffaloes whose semen is produced at the National Artificial Insemination Center are distributed in various regions in Indonesia. In addition, inbreeding reduction and genetic improvement can be performed using AI. The government has implemented a national program called “SIKOMANDAN” to increase the productivity of cattle and buffaloes. Selected buffalo breeds in Indonesia are produced by the Center for Superior Livestock Breeding and Forage Animal Feed (BPTU-HPT) Siborongborong, which is one of the Technical Implementation Units in Indonesia involved in the production, development, and distribution of buffalo superior breeding stocks. This UPT can be a supplier of excellent buffalo breeding stock to improve the quality of buffalo available to farmers. The genetic quality of buffaloes must be improved with the support of all relevant stakeholders, including research institutions, local governments, and universities.

The genetic quality of buffaloes in Indonesia can be improved with the following objectives:

1. Improved milk production: Milk production is an important source of income for smallholder farmers in Indonesia. Genetic selection can be used to identify buffaloes with higher milk yields and better milk quality.
2. Increasing reproduction efficiency: Improving reproductive efficiency is crucial for the sustainable development of the buffalo industry in Indonesia. Genetic selection can be used to identify buffaloes with desirable reproductive traits, such as early sexual maturity and higher conception rates, reducing calving intervals, and increasing productivity.
3. Improving meat quality: Meat from buffalo is highly valued in Indonesia. Genetic selection can help identify animals with desirable meat quality traits, such as marbling, tenderness, and flavor, improving the value and marketability of buffalo meat [91, 92].

Data management systems and software applications can support the genetic improvement of buffaloes at BPTU-HPT. These systems allow data collection, storage, and analysis on animal performance, pedigree, and genetic markers. Such systems can provide valuable insights into the genetic potential of buffaloes and help make informed decisions about breeding and selection. Genomic selection can be used for the genetic improvement of buffaloes. Genomic selection is a modern breeding technique that involves using DNA markers to predict the genetic potential of an animal for specific traits. Genomic selection technology has been extensively used to obtain cattle with superior economic traits; however, this technology has not been widely used for buffalo. In buffaloes, genomic selection can be used to identify animals with desirable traits, such as superior milk yield and disease resistance.

## Conclusion

There is an immediate need to map actual field conditions regarding buffalo distribution in Indonesia and provide clarity regarding breeds. To implement targeted breeding programs, it is essential to lay the groundwork for understanding the distribution of specific traits and the genetic makeup of distinct buffalo breeds. Indonesian indigenous buffalo breeds have traditionally been classified based on morphological distinctions. However, geographical variances are insufficient to identify closely related breeds. Therefore, precise identification and evaluation of distinctions among breeds using genetics and genomics is essential to improve buffalo breeding and conservation. Indonesia must create long-term planning measures and designate organizations to ensure that resources in various agroecological zones are harnessed responsibly to increase buffalo productivity.

## Authors’ Contributions

PWP: Conceptualization, literatures search, figure design, drafting the manuscript, and revision. YAT: Conceptualization, literatures search, data collection and analysis, drafting the manuscript, and revision. DNHH: Conceptualization, literatures search, drafting the manuscript, review, and revision. APZNLS: Conceptualization, literatures search, data collection and analysis, drafting the manuscript, and revision. LP: Literatures search, figure design, and drafting the manuscript. EH: Literatures search, figure design, drafting the manuscript, revision, and proofreading. BT: Literatures search, drafting the manuscript, review, supervision, and proofreading. ER: Review, supervision, and revision. PRM and AW: Supervision, revision, and proofreading. All authors have read, reviewed, and approved the final manuscript.

## References

[1] Statistics Indonesia (2022). Peternakan Dalam Angka.

[2] Borghese A, Mazzi M (2005). Buffalo population and strategies in the world. In:Buffalo Production and Research, REU Technical Series. Food and Agricultural Organization, Rome.

[3] Colli L, Milanesi M, Vajana E, Iamartino D, Bomba L, Puglisi F, Del Corvo M, Nicolazzi E.L, Ahmed S.S.E, Herrera J.R.V, Cruz L, Zhang S, Liang A, Hua G, Yang L, Hao X, Zuo F, Lai S.J, Wang S, Liu R, Gong Y, Mokhber M, Mao Y, Guan F, Vlaic A, Vlaic B, Ramunno L, Cosenza G, Ahmad A, Soysal I, Ünal E.Ö, Ketudat-Cairns M, Garcia J.F, Utsunomiya Y.T, Baruselli P.S, Amaral M.E.J, Parnpai R, Drummond M.G, Galbusera P, Burton J, Hoal E, Yusnizar Y, Sumantri C, Moioli B, Valentini A, Stella A, Williams J.L, Ajmone-Marsan P (2018). New insights on water buffalo genomic diversity and post-domestication migration routes from medium density SNP chip data. Front. Genet.

[4] Deb G.K, Nahar T.N, Duran P.G, Presicce G.A (2016). Safe and sustainable traditional production:The water buffalo in Asia. Front. Environ. Sci.

[5] Macgregor R (1941). The domestic buffalo. Vet. Rec.

[6] Wahid H, Rosnina Y (2016). Buffalo:Asia. Reference Module in Food Science B978-0-08-100596-5.21231-6.

[7] Beaton W.G (1974). The husbandry and health of the domestic buffalo. Trop. Anim. Health Prod.

[8] Zhang Y, Colli L, Barker J.S.F (2020). Asian water buffalo:Domestication, history and genetics. Anim. Genet.

[9] Minervino A.H.H, Zava M, Vecchio D, Borghese A (2020). *Bubalus bubalis*:A short story. Front. Vet. Sci.

[10] Zhang Y, Lu Y, Yindee M, Li K.Y, Kuo H.Y, Ju Y.T, Ye S, Faruque M.O, Li Q, Wang Y, Cuong V.C, Pham L.D, Bouahom B, Yang B, Liang X, Cai Z, Vankan D, Manatchaiworakul W, Kowlim N, Duangchantrasiri S, Wajjwalku W, Colenbrander B, Zhang Y, Beerli P, Lenstra J.A, Barker J.S.F (2016). Strong and stable geographic differentiation of swamp buffalo maternal and paternal lineages indicates domestication in the China/Indochina border region. Mol. Ecol.

[11] Nagarajan M, Nimisha K, Kumar S (2015). Mitochondrial DNA variability of domestic river buffalo (*Bubalus bubalis*) populations:Genetic evidence for domestication of river buffalo in Indian subcontinent. Genome Biol. Evol.

[12] Zhang Y, Vankan D, Zhang Y, Barker J.S.F (2011). Genetic differentiation of water buffalo (*Bubalus bubalis*) populations in China, Nepal and South-East Asia:Inferences on the region of domestication of the swamp buffalo. Anim. Genet.

[13] Cockrill W.R (1981). The water buffalo:A review. Br. Vet. J.

[14] Sun T, Wang S, Chanthakhoun V, Dang R, Huang Y, Chen H, Lei C (2020). Multiple domestication of swamp buffalo in China and South East Asia. J. Anim. Breed. Genet.

[15] Baan A, Allo M.D.G, Patak A.A (2022). The cultural attitudes of a funeral ritual discourse in the indigenous Torajan, Indonesia. Heliyon.

[16] Hardjosubroto W (2006). Kerbau Mutiara Yang Terlupakan Dalam Peternakan. Orasi lmiah Purna Tugas, Yogyakarta.

[17] DAD-IS Domestic Animal Diversity Information System.

[18] Marai I.F.M, Haeeb A.A.M (2010). Buffalo's biological functions as affected by heat stress-a review. Livest. Sci.

[19] Talib C, Herawati T, Hastono H (2014). Strategies for increasing buffalo productivity through improvement in feed and genetic. Wartazoa.

[20] Sumantri C, Gunawan A, Anggraeni A (2021). Karakteristik Genetik Kerbau Lokal dan Prospek Pengembangannya. IPB Press, Bogor.

[21] Cruz L (2013). Changing faces of swamp buffaloes in an industrializing Asia. Buffalo Bull.

[22] Ditjenpkh (2022). Livestock and Animal Health Statistics 2022. Direktorat Jenderal Peternakan dan Kesehatan Hewan, Kementerian Pertanian RI, Jakarta.

[23] Hegde N.G (2019). Buffalo husbandry for sustainable development of small farmers in India and other developing countries. Asian J. Res. Anim. Vet. Sci.

[24] Ikun A (2018). Factors influencing buffalo population levels in Biboki Anleu District, North Central Timor Regency [Faktor-faktor yang mempengaruhi tingkat populasi ternak kerbau di Kecamatan Biboki Anleu Kabupaten Timor Tengah Utara]. JAS.

[25] Ernest E.M, Haanes H, Bitanyi S, Fyumagwa R.D, Msoffe P.L, Bjørnstad G, Røed K.H (2012). Influence of habitat fragmentation on the genetic structure of large mammals:Evidence for increased structuring of African buffalo (*Syncerus caffer*) within the Serengeti ecosystem. Conserv. Genet.

[26] Metzger K.L, Sinclair A.R.E, Hilborn R.J, Hopcraft J.G.C, Mduma S.A.R (2010). Evaluating the protection of wildlife in parks:The case of African buffalo in Serengeti. Biodivers. Conserv.

[27] Mureithi S.M, Verdoodt A, Njoka J.T, Olesarioyo J.S, Ranst E.V, Kideghesho J.R, Rija A.A (2019). Community-based conservation:An emerging land use at the livestock-wildlife interface in Northern Kenya. Wildlife Management-failures, Successes and Prospects. IntechOpen, Rijeka.

[28] Suhardi S, Summpunn P, Duangjinda M, Wuthisuthimethavee S (2020). Phenotypic diversity characterization of Kalang and Thale Noi Buffalo (*Bubalus bubalis*) in Indonesia and Thailand:Perspectives for the buffalo breeding development. Biodiversitas.

[29] Rusli R, Syahidin S (2021). Characteristics of breeders and development strategies for Gayo buffalo livestock in the Peruweren system [Karakteristik peternak dan strategi pengembangan ternak kerbau Gayo sistem peruweren]. J. Ilmu Teknol. Peternakan.

[30] Rias M.I, Putra R.A, Madarisa F (2020). Study of the buffalo farming business management system in Sijunjung Regency [Kajian sistem pengelolaan usaha peternakan kerbau di Kabupaten Sijunjung]. Agrifo J. Agribisnis Univ. Malikussaleh.

[31] Kartika K, Sirajuddin S.N, Rasyid I (2016). Factors affecting low buffalo ownership in Sumbang village, Curio sub district, Enrekang district. J. Ilmu Teknol. Peternakan.

[32] Widi T.S.M, Pratowo S, Sulaiman A, Hulfa R, Sumantri I (2021). Reproductive characteristics of female swamp buffalo reared under Kalang production system in South Kalimantan. IOP Conf. Ser. Earth Environ. Sci.

[33] Suhaimi A, Zarmiyeni Z, Saihani A, Van Royensyah R (2016). The comparative advantage and development strategy assesment of swamp bufallo in South Kalimantan. Rawa Sains J. Sains Stiper Amuntai.

[34] Rusli R, Nikmah A, Ridhana F, Sufian S (2021). Study of Gayo buffalo livestock development and development strategies in Linge District, Central Aceh Regency [Kajian pengembangan ternak kerbau Gayo serta strategi pengembangannya di Kecamatan Linge Kabupaten Aceh Tengah]. J. Ilmu Peternakan Vet.

[35] Komariah K, Kartiarso K, Lita M (2014). Productivity of swamp buffalo at district of Muntai, Kutai Kartanegara of East Kalimantan province. Bul. Peternakan.

[36] Kusnadi A, Kusumaningrum D.A, Sianturi R.G, Triwulaningsih E (2005). Fungsi dan Peranan Kerbau dalam Sistem Usaha Tani di Provinsi Banten. In:Prosiding Seminar dan Lokakarya Nasional Kerbau:Potensi Kerbau menunjang Kecukupan Daging Nasional. Pusat Penelitian dan Pengembangan Peternakan, Bogor.

[37] Abd El-Salam M.H, El-Shibiny S (2011). A comprehensive review on the composition and properties of buffalo milk. Dairy Sci. Technol.

[38] Garau V, Manis C, Scano P, Caboni P (2021). Compositional characteristics of Mediterranean buffalo milk and whey. Dairy.

[39] Abesinghe A.M.N.L, Priyashantha H, Prasanna P.H.P, Kurukulasuriya M.S, Ranadheera C.S, Vidanarachchi J.K (2020). Inclusion of probiotics into fermented buffalo (*Bubalus bubalis*) milk:An overview of challenges and opportunities. Fermentation.

[40] Vargas-Ramella M, Pateiro M, Maggiolino A, Faccia M, Franco D, De Palo P, Lorenzo J.M (2021). Buffalo milk as a source of probiotic functional products. Microorganisms.

[41] OECD Southeast Asia:Prospects and challenges. In:OECD-FAO Agricultural Outlook 2017–2026. OECD Publishing, Paris.

[42] FAOSTAT about Live Animals, Data on Buffaloes.

[43] Komariah K, Burhanuddin B, Dzaki M, Aditia E.L, Mendrofa V.A (2020). Performance and development strategy for swamp buffalo (*Bubalus bubalis*) in Serang district Indonesia. J. Ilmu Prod. Teknol. Hasil Peternakan.

[44] Rusdiana S, Praharani L, Sianturi R.G (2020). The economic efficiency of buffalo business (*Bubalus bubalis*) with the shepherd. SOCA J. Sos. Ekon. Pertanian.

[45] Budiarto A, Ciptadi G (2018). The productivity and natural increase of swamp buffalo in district Malang. IOP Conf. Ser. Earth Environ. Sci.

[46] Nafianda N, Panjuni M.M, Pratiwi H, Awaludin A, Rukmi D.L, Syahniar T.M (2021). Kajian Karakteristik Peternak Terhadap Tingkat Kebuntingan Sapi Potong di Kecamatan Nusawungu, Cilacap, Jawa Tengah. In:The 2^nd^ Conference of Applied Animal Science. Politeknik Negeri Jember, Jember.

[47] Suryanto B, Arifin M, Rianto E (2002). Potential of swamp buffalo development in Central Java, Indonesia. Buffalo Bull.

[48] Wirawati C.U, Sudarwanto M.B, Lukman D.W, Wientarsih I (2017). Characteristic and development of cow's milk dadih as an alternate of buffalo's milk dadih [Karakteristik dan pengembangan dadih dari susu sapi sebagai alternatif dadih susu kerbau]. Wartazoa.

[49] Sulmiyati S, Said N.S (2018). Characteristics of dangke derived from buffalo milk with addition of dry crude papain [Karakteristik dangke susu kerbau dengan penambahan crude papain kering]. Agritech.

[50] Diana D, Aritonang E.Y, Purba A, Lubis R (2020). Utilization of local food to overcome nutritional problems among toddlers in Medan Tuntungan. Adv. Health Sci. Res.

[51] Marhama M, Saloko S, Cicilia S (2022). The effect of substitution of buffalo milk with soy milk on the nutritional value of palopo [Pengaruh susbtitusi susu kerbau dengan susu kedelai terhadap nilai gizi palopo]]. J. Food Agric. Prod.

[52] Mesloy M.H, Refli R, Mauboy R.S (2019). Level of *Escherichia coli* contamination in traditional Moa cheese with different pasteurization durations [Tingkat cemaran *Escherichia coli* pada keju tradisional Moa dengan durasi pasteurisasi berbeda]. J. Biotrop. Sains.

[53] Melia S, Yuherman Y, Jaswandi J, Purwati E (2018). Selection of buffalo milk lactic acid bacteria with probiotic potential. Asian J. Pharm. Clin. Res.

[54] Rizqiati H, Nurwantoro N, Susanti S, Surya K.R.A, Prayoga M.I.Y (2021). Chemical and microbiological properties of buffalo milk kefir with different starter concentrations. IOP Conf. Ser. Earth Environ. Sci.

[55] Alang H, Kusnadi J, Ardyati T, Suharjono S (2020). Nutrition characteristics of Buffalo milk from Toraya, Makassar [Karakteristik nutrisi susu kerbau Belang Toraja, Makassar]. Zootec.

[56] Susmiati S, Melia S, Purwati E, Alzahra H (2022). Physicochemical and microbiological fermented buffalo milk produced by probiotic *Lactiplantibacillus pentosus* HBUAS53657 and sweet orange juice (*Citrus nobilis*). Biodiversitas.

[57] Thamrin M.H, Ismanida I, Handayani M (2018). Utilization of buffalo milk curd to provide functional supplementary food (PMT) for toddlers [Pemanfaatan dadih susu kerbau untuk pemberian makanan tambahan (PMT) fungsional anak balita]. J. Sehat Mandiri.

[58] Girsang V.I, Damanik E, Tampubolon L.F, Harianja E.S (2023). Education about the benefits of dali ni horbo in preventing stunting [Edukasi tentang manfaat dali ni horbo dalam penangulangan stunting]. J. Abdimas Mutiara.

[59] Arfiani A, Fuah A.M, Salundik S, Purwanto B.P (2015). The motivation and participation of farmers in the farming development buffaloes in Pandeglang District (case study:Cibarani Village Cisata Sub-District [Motivasi dan partisipasi peternak dalam pengembangan ternak kerbau di Kabupaten Pandeglang (studi kasus:Desa Cibarani Kecamatan Cisata)]. J. Sains Terapan.

[60] Elizabeth R (2017). Strengthening and developing buffalo through the empowerment of breeder group to sufficient meat consumption needs in Indonesia [Penguatan dan pengembangan ternak kerbau melalui pemberdayaan kelompok peternak dalam memenuhi kebutuhan konsumsi daging di Indonesia]. UNES J. Scientech Res.

[61] Mufiidah N, Ihsan M.N, Nugroho H (2013). The productivity of female swamp buffaloes *(Bubalus bubalis carabanesis*) in terms of reproductive performance and body measurements at Tempursari Subdistrict Lumajang Regency [Produktivitas induk kerbau rawa *(Bubalus bubalis*) ditinjau aspek kinerja reproduksi dan ukuran tubuh di Kecamatan Tempursari Kabupaten Lumajang]. Ternak Trop.

[62] Rehman S.U, Hassan F.U, Luo X, Li Z, Liu Q (2021). Whole-genome sequencing and characterization of buffalo genetic resources:Recent advances and future challenges. Animals (Basel).

[63] García-Dorado A, Caballero A (2021). Neutral genetic diversity as a useful tool for conservation biology. Conserv. Genet.

[64] Sikdar S Das, T, Sajib E.H, Rahman K.M.U, Siddiki A.Z, Uddin M.B (2021). Multi-OMICS and molecular biology perspective in buffalo genome. J. Buffalo Sci.

[65] Wolstenholme D.R (1992). Animal mitochondrial DNA:Structure and evolution. Int. Rev. Cytol.

[66] Brown W.M, Prager E.M, Wang A, Wilson A.C (1982). Mitochondrial DNA sequences of primates:Tempo and mode of evolution. J. Mol. Evol.

[67] Sukri A, Amin M, Winaya A, Gofur A (2015). Substitution and haplotype diversity analysis on the partial sequence of the mitochondrial DNA cyt *b* of Indonesian swamp buffalo (*Bubalus bubalis*). Biol. Med. Nat. Prod. Chem.

[68] Rusdin M, Solihin D.D, Gunawan A, Talib C, Sumantri C (2020). Genetic variation of eight Indonesian swamp-buffalo populations based on cytochrome b gene marker. Trop. Anim. Sci. J.

[69] Suhardi S, Summpunn P, Wuthisuthimethavee S (2021). MtDNA D-loop sequence analysis of Kalang, Krayan, and Thale Noi buffaloes (*Bubalus bubalis*) in Indonesia and Thailand reveal genetic diversity. J. Indones. Trop. Anim. Agric.

[70] Priyono D.S, Solihin D.D, Farajallah A, Arini D.I.D (2018). Anoa, dwarf buffalo from Sulawesi, Indonesia:Identification based on DNA barcode. Biodiversitas.

[71] Amin M, Suarsini E, Azmi I, Gofur A (2016). Phylogenetic analysis of local endemic buffalo (*Bubalus bubalis*) based on cytochrome B gene in central Indonesia. J. Teknol.

[72] Winaya A, Sukri A, Gofur A, Amin M (2019). The genetic divergence and phylogenetic relationship of Indonesia swamp buffalo (*Bubalus bubalis*) based on partial sequences of cytochrome B gene of mitochondrial DNA. Int. J. Eng. Technol.

[73] Stange M, Barrett R.D.H, Hendry A.P (2021). The importance of genomic variation for biodiversity, ecosystems and people. Nat. Rev. Genet.

[74] Kardos M, Armstrong E. E, Fitzpatrick S. W, Hauser S, Hedrick P. W, Miller J. M, Tallmon D.A, Funk W. C (2021). The crucial role of genome-wide genetic variation in conservation. Proc. Natl. Acad. Sci. U. S. A.

[75] Kumar B, Sahoo A.K, Dayal S, Das A.K, Taraphder S, Batabyal S, Ray P.K, Kumari R (2019). Genetic profiling of Hsp70 gene in Murrah buffalo (*Bubalus bubalis*) under sub-tropical climate of India. Cell Stress Chaperones.

[76] Bora A, Bora P, Majumdar S, Singh N.S, Kalita G, Behera P, Mayengbam P, Tolenkhomba T.C (2021). Genetic polymorphism of melatonin receptor (MTNR1A) gene in swamp buffalo of Assam and Manipur, India. Int. J. Livest. Res.

[77] Kathiravan R.S, Vandana C.M, Malarmathi M, Chitra R, Murali N, Arthanarieswaran M (2021). A study on b-casein gene polymorphism in crossbred cattle and Murrah/graded Murrah buffalo in Tamil Nadu. Indian J. Anim. Res.

[78] Dinesh K, Verma A, Gupta I.D (2021). Lactoferrin gene polymorphism of exons 8 and 13 in Murrah buffalo. Indian J. Dairy Sci.

[79] Sumantri C, Anggraeni A, Sari E.M, Andreas E (2013). Genetic Polymorphism of Growth Hormone Genes in Indonesian Local Buffalo. In:Proceeding of the 4^th^ International Conference on Sustainable Animal Agriculture for Developing Countries, Lanzhou.

[80] Eriani K, Rahmi R, Jamil I, Rosnizar R, Azhar A (2019). Body size characteristics and polymorphism in GH and GHRH genes of Simeulue Buffalo of Aceh, Indonesia. Biodiversitas.

[81] Wahyuni V, Nafiu L.O, Pagala M.A (2016). Phenotypic characteristics of qualitative and quantitative traits of Kacang goats in West Muna Regency [Karakteristik fenotip sifat kualitatif dan kuantitatif kambing Kacang di Kabupaten Muna Barat]. J. Ilmu Teknol. Peternakan Trop.

[82] Misrianti R, Sumantri C, Farajallah A (2010). Polymorphism identification of pit1 gene in Indonesian buffaloes (*Bubalus Bubalis*) and Holstein-Friesian cows. Media Peternakan.

[83] Rini A.O, Sumantri C, Damayanthi E (2014). k-casein gene polymorphisms in riverine and swamp buffalo in Indonesia. J. Indones. Trop. Anim. Agric.

[84] Margawati E.T, Volkandari S.D, Indriawati I, Talib C (2017). Genotyping of Kappa-Casein gene of buffalo in Indonesian. In:Proceedings of International Seminar on Livestock Production and Veterinary Technology.

[85] Gurao A, Vasisth R, Singh R, Dige M.S, Vohra V, Mukesh M, Kumar S, Kataria R.S (2022). Identification of differential methylome signatures of white pigmented skin patches in Nili Ravi buffalo of India. Environ. Mol. Mutagen.

[86] Saputra S, Dinoto A (2021). Cellulolytic and xylanolytic faecal bacteria from tedong bonga, [Toraja buffalo, *Bubalus bubalis carabanesis*]. IOP Conf. Ser. Earth Environ. Sci.

[87] Yusnizar Y, Wilbe M, Herlino A.O, Sumantri C, Noor R.R, Boediono A, Andersson L, Andersson G (2015). Microphthalmia-associated transcription factor mutations are associated with white-spotted coat color in swamp buffalo. Anim. Genet.

[88] Saputra F, Jakaria J, Anggraeni A, Sumantri C (2020). Genetic diversity of Indonesian swamp buffalo based on microsatellite markers. Trop. Anim. Sci. J.

[89] Saputra F, Anggraeni A (2023). Morphometric diversity of swamp buffaloes in Indonesia:A meta-analysis. Buffalo Bull.

[90] Hariyono D.N.H (2022). Application of microsatellite markers for genetic diversity analysis of Indonesian local cattle. Wartazoa.

[91] Prihandini P.W, Primasari A, Aryogi A, Efendy J, Luthfi M, Pamungkas D, Hariyono D.N.H (2021). Genetic variation in the first intron and exon of the myostatin gene in several Indonesian cattle populations. Vet. World.

[92] Prihandini P.W, Hariyono D.N.H, Tribudi Y.A (2021). Myostatin gene as a genetic marker for growth and carcass traits in beef cattle. Wartazoa.

